# Advancements in combining electronic animal identification and augmented reality technologies in digital livestock farming

**DOI:** 10.1038/s41598-023-45772-2

**Published:** 2023-10-25

**Authors:** Daniele Pinna, Gabriele Sara, Giuseppe Todde, Alberto Stanislao Atzori, Valentino Artizzu, Lucio Davide Spano, Maria Caria

**Affiliations:** 1 Department of Agricultural Sciences, University of Sassari, Viale Italia 39/A, 07100 Sassari, Italy; 2 Department of Mathematics and Computer Science, University of Cagliari, Via Ospedale 72, 09124 Cagliari, Italy

**Keywords:** Engineering, Information technology, Software

## Abstract

Modern livestock farm technologies allow operators to have access to a multitude of data thanks to the high number of mobile and fixed sensors available on both the livestock farming machinery and the animals. These data can be consulted via PC, tablet, and smartphone, which must be handheld by the operators, leading to an increase in the time needed for on-field activities. In this scenario, the use of augmented reality smart glasses could allow the visualization of data directly in the field, providing for a hands-free environment for the operator to work. Nevertheless, to visualize specific animal information, a connection between the augmented reality smart glasses and electronic animal identification is needed. Therefore, the main objective of this study was to develop and test a wearable framework, called SmartGlove that is able to link RFID animal tags and augmented reality smart glasses via a Bluetooth connection, allowing the visualization of specific animal data directly in the field. Moreover, another objective of the study was to compare different levels of augmented reality technologies (assisted reality vs. mixed reality) to assess the most suitable solution for livestock management scenarios. For this reason, the developed framework and the related augmented reality smart glasses applications were tested in the laboratory and in the field. Furthermore, the stakeholders’ point of view was analyzed using two standard questionnaires, the NASA-Task Load Index and the IBM-Post Study System Usability Questionnaire. The outcomes of the laboratory tests underlined promising results regarding the operating performances of the developed framework, showing no significant differences if compared to a commercial RFID reader. During the on-field trial, all the tested systems were capable of performing the task in a short time frame. Furthermore, the operators underlined the advantages of using the SmartGlove system coupled with the augmented reality smart glasses for the direct on-field visualization of animal data.

## Introduction

In recent decades, many technologies have been introduced into the livestock farming sector. Among these, one of the earliest technologies was radio frequency identification (RFID), which is used for the electronic identification (EID) of animals^1^. RFID systems are composed of two parts, a transponder or tag (ear tags, rumen bolus, or injectable glass tags) and a transceiver (portable or fixed)^2^. The use of EID is mandatory in Europe for sheep and goats (Reg. CE n. 21/2004), while it is voluntary for cattle. RFID tags can use two communication protocols, half-duplex (HDX) and full-duplex (FDX). As described in ISO 11785:1996^3^, these two technologies differ in the modulation of the response signal, return frequencies, encoding and bit rate of transmission. However, even if the response telegram structure differs for HDX and FDX systems, the structure of the unique animal code (64 bits) is the same and is regulated by ISO 11784:1996^4^. Even if this technology is considered established for identification, tags can only store a few bits (128 bits in FDX and 112 bits in HDX), which correspond to the unique identification code of the animal, giving no additional information. Moreover, a large variety of data are collected in modern farms from different sensors thanks to the spread of precision livestock farming (PLF) technologies. The most common definition of PLF is “individual animal management by continuous real-time monitoring of health, welfare, production/reproduction, and environmental impact”^5^. However, PLF is not the only term used to describe this kind of approach to livestock farming; among the others, "smart livestock farming” and "smart animal agriculture” are the most commonly used^6^. All these terms refer to the use of process engineering principles or technologies to manage livestock production through smart sensors, monitoring animal growth, production, diseases, behavior and components of the macroenvironment^7^. PLF Sensors can be fixed (e.g., cameras or weather station) or wearable by the animal (e.g., bolus, collars or ear tags)^8^. Fixed sensors, especially cameras, can be used for non-invasive monitoring of animals, thanks to the use of machine vision. This technology can provide very specific and precise information concerning animal behavior and health status, but the use for individual identification still requires further investigation^9^. In general, PLF technologies have made available a large amount of data; however, their consultation and interpretation by farmers is often considered a difficult and time-consuming task^10^. Often, the interoperability between different sensors is limited and data are stored in different databases accessible through PCs or mobile devices, such as smartphones and tablets. However, the visual presentation of raw or summarized data, especially directly on-field, represents a crucial part for an effective use of sensors outcomes^11^. Moreover, even when the data can be consulted through mobile devices, the process implies a stop in normal farm management activities because the smartphone or the tablet occupies the operator’s hand.

In this scenario, smart glasses for augmented reality (ARSG), if connected to EID, could allow the possibility of consulting specific animal databases directly on-field, leaving the operator hands-free^12^. ARSGs are wearable head-up displays, connected to, or integrating a miniaturized computer, that adds virtual information to the user’s reality. There are many types of ARSG that can be grouped according to price, weight, powering system (internal or external battery), visualization systems (video, optical or retinal), operating system (Android-based, Windows, etc.), interaction methods and resistance to bumps, dust and water^13^. Augmented Reality (AR) consists of the visualization of digital information superimposed on the real environment, providing additional information to users and helping them to solve tasks at the same time^14^. At this moment, AR is not a diffuse technology in the agricultural domain but is more commonly used in manufacturing^15–17^, industrial sectors^18,19^, medicine^20,21^, psychology^22,23^ and education^24–26^. Several studies have already shown that AR can be a useful technology in agricultural contexts^27,28^. Azuma (1997)^29^ defined the three basic characteristics of the AR system and expanded the definition in 2001. However, in recent years, with the advancement of technology and the diversification of devices that can implement AR technology, a new definition of AR was needed. In fact, Rauschnabel et al.^30^ redefined AR as a hybrid experience consisting of context-specific virtual content that is merged into a user’s real-time perception of the physical environment through computing devices. AR can further be refined based on the level of integration of the digital elements in the real world. The level of integration defines a specific AR spectrum that ranges from assisted reality (low integration) to mixed reality (high integration). This more comprehensive definition allows us to include a wider variety of technologies in the AR spectrum, as in the case of assisted reality. This technology consists of the visualization of head-stable content not connected to real-world objects, and it is commonly implemented in mobile devices, such as smartphones, and in smart glasses (SG). Another level of the AR spectrum is mixed reality (MR), which was first described by Milgram and Kishino^31^ as “a subclass of virtual reality (VR) related technologies that involve the merging of the real and virtual worlds”. Different from assisted reality systems, MR increases the user’s spatial and visual interaction possibilities^32^. MagicBook is considered one of the first examples of an MR system^33^. This book can be read and held as a normal one but with a specific MR display a set of digital information, and 3D models, aligned with the real-world book, are shown. The device also allows the user to be immersed in the virtual scene, exploring the entire virtual continuum spectrum with one system. Currently, one of the most advanced MR devices is the Microsoft HoloLens (Microsoft, USA), which has the capability to show digital information and virtual objects in the form of holograms. Those objects are aligned to the real world thanks to real-time environment scanning and can interact with the use of bare hands. The principal situation in which it is better to choose MR over AR is when there is the need to manipulate and physically interact with virtual objects^34^.

The aim of this study was to design and develop a framework that allows the connection of animal electronic identification to different types of ARSG and to evaluate the performance of the developed system in the laboratory and on-field. Moreover, a comparison of assisted and mixed reality systems was carried out to assess the most suitable solution in livestock farm environments.

## Developed system framework

### SmartGlove hardware

**Figure 1:**
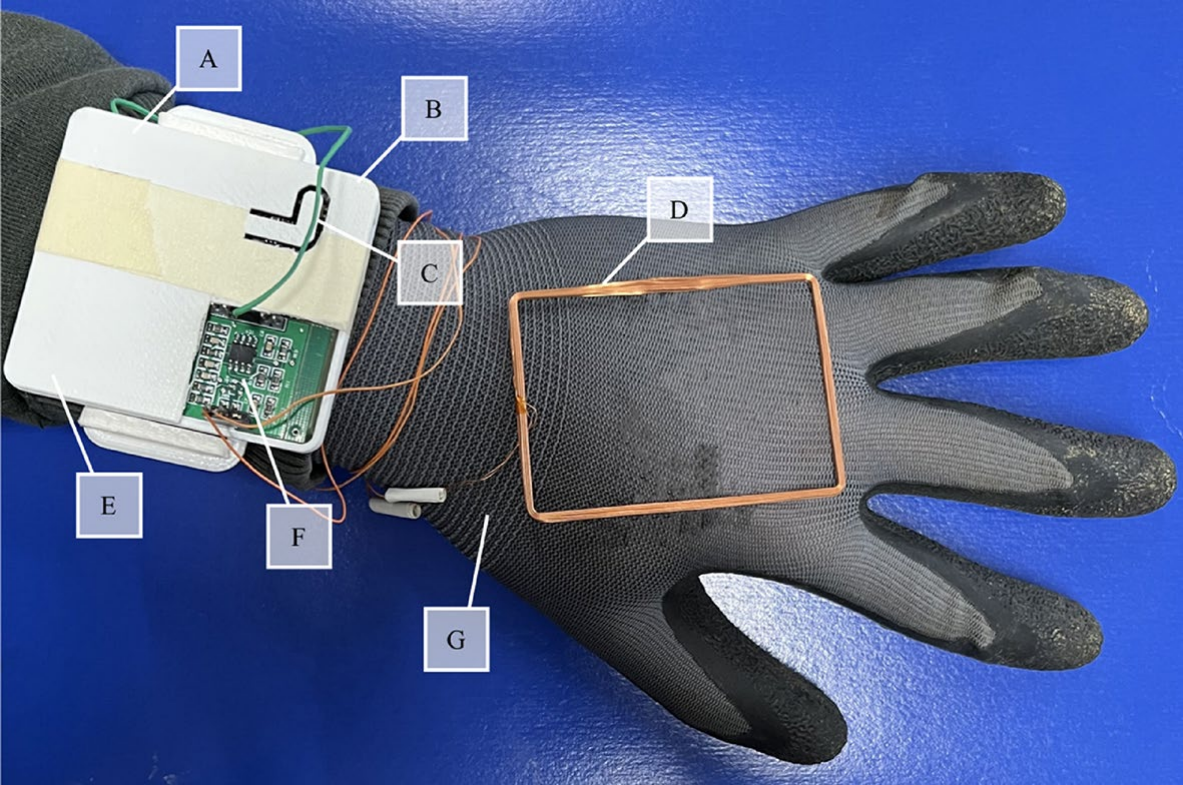
SmartGlove hardware components: (**A**) 3.7 V 2000mAh Li-Ion battery, (**B**) 3D printed case, (**C**) connection status led, (**D**) 125 kHz copper antenna, (**E**) Arduino Bluetooth motherboard, (**F**) FDX-B RFID reader controller, (**G**) support glove.

SmartGlove (SMGL; Fig. 1) is currently a TRL-3 (technology readiness level-3: analytical and experimental critical function and/or characteristic proof of concept) prototype that allows reading the unique animal code from RFID tags and sending it to ARSG to display all the information related to that specific animal. It is composed of an Arduino control unit with integrated Bluetooth and an RFID reader board connected to a 125 kHz antenna. All the components are enclosed in a 3D-printed plastic case that can be used as a bracelet, with the antenna extended in the back of the hand attached to a glove (Fig. 1).

The SMGL is connected, via Bluetooth, to custom software for SG, which displays the following animal information related to the tag’s identification code: The animal ID code, group (A or B), name, date of the last parturition, age (in years), milk production of the last day (kg), milk production of the last week (kg), the number of parturitions and presence of mastitis (P for positive or N for negative). The SMGL can be connected to different types of ARSG. The first ARSG adopted in this study are the Epson Moverio BT–300 (BT300), an Android-based assisted reality device with an optical, binocular, see-through display. The second ones are the Microsoft HoloLens 2 (HL), a Microsoft Windows-based MR device, which has a holographic display. A complete list of the characteristics of both SG can be found in Table 1.

**Table 1:** Technical characteristics of the HoloLens 2 (HL) and Moverio BT-300 (BT300).

Characteristic	HL	BT300
Manufacturer	Microsoft	Epson
Processor	Qualcomm Snapdragon 850 (8 core 2.95 GHz)	Intel Atom (4 core 1.44 GHz)
Operating system	Windows Holographic	Moverio OS (Android 5.1.1)
Display	See-through holographic lenses	Binocular Si-OLED
RAM	4 GB	2 GB
Weight	566 g	69 g
Battery duration	3 h	4 h
Controller input	Hand/voice recognition	Joypad (touchpad)

### Software for Smart Glasses

To design and develop the supporting applications that run on the BT-300 and HL devices we used Android SDK for the former and Universal Windows Platform (UWP) for the latter. Both SDKs enable the developer to create the same experience for their respective target devices with no noticeable difference in the use of the resulting applications. For both application versions the same implementation architecture has been adopted, so the main differences between them are platform-dependent and at a code level.

The development environments enabled us to create the same user interface in both the resulting applications. Indeed, both APIs share some common design practices:

- The management of the sensors of the device is handled by a Hardware Abstraction Layer, that gives the developer the possibility to interact with them without knowing how to access them directly;
- It is possible to visually design the interfaces using the built-in tools in the Integrated Development Environment applications.
- Database management can be handled in the same way since both of the APIs implement the same database engine (SQLite).
- Before accessing any device features both APIs need the developer to declare beforehand which features the application will ask authorization from the user (either on application install or at runtime)

Despite these similarities, the APIs differs in some other features:

- the programming languages are different (Android SDK supports Java and Kotlin for the majority of the use cases, with the possibility to use C++ as a performance-critical, native language; UWP supports C#, Visual Basic and C++ with WinRT as possible alternatives)
- The operating systems the APIs target are different (Android SDK targets Android devices, UWP targets Windows devices).
- the user interface, while it can be designed in visual ways from both SDKs, it requires different markup languages for customizing them using the underlying code (Android SDK uses XML-based layouts, UWP uses XAML-based pages).
- The notification system includes on-screen popout messages (“toast notifications”), bottom application messages (“Snackbar notifications”) or system notifications on Android SDK, and only system notifications on UWP.

Moreover, strictly speaking about the target devices, interaction techniques and visualization of the interface itself differs in a key difference: while BT-300 shows a semi-transparent Android interface in front of the user, it is stationary with respect to the movement of head of the user and it can be interacted with a touchscreen joypad, HL uses a billboarding technique for keeping the interface in a 3D space (with the possibility to enable a "tag-along" feature for moving the interface with the user), and it can be interacted using air gestures and voice recognition.

### Software implementation architecture

**Figure 2:**
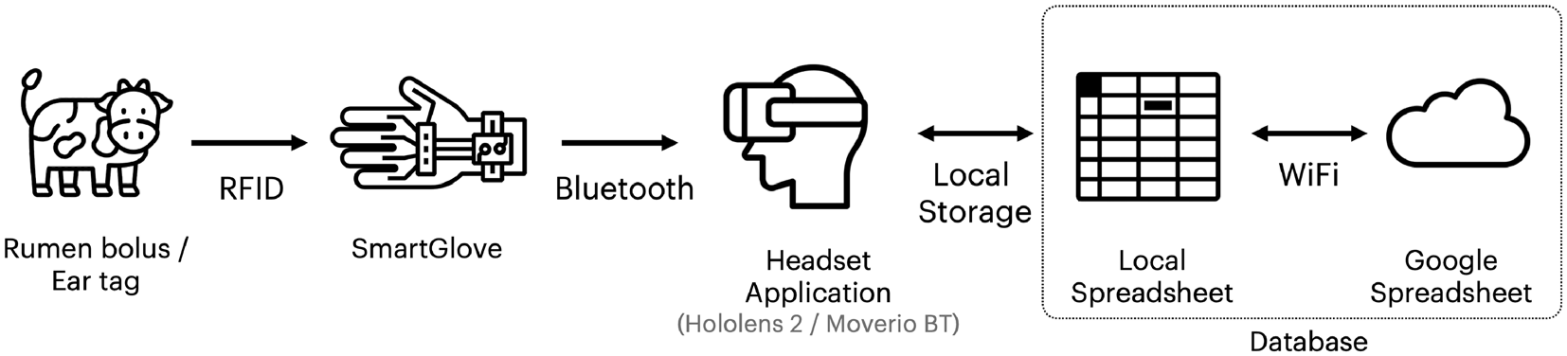
Overview of the software solution for smart glasses.

The software solution consists of three modules (Fig. 2), the glove hardware manager, the database and the headset application. The SMGL is controlled through an Adafruit board connected to an RFID antenna, which reads rumen bolus or ear tags to recognize the animal and sends the identifier to the headset through a Bluetooth connection. When the interface receives a new identifier, it displays all the information about the animal stored in the database.

In the current prototype, the database structure is straightforward. It consists of a Google spreadsheet farmers can modify to adapt to their needs. This means they can add the information they would like to store about the animals by adding columns to the spreadsheet. The only assumption is that the RFID identifier is the first column in the spreadsheet, serving as the primary key. We can upload a copy of the shared spreadsheet in the interface application as Comma Separated Values (CSV) for offline work, and synchronize the changes when the network is available. The categories are not fixed in the code. Indeed, they are defined by the CSV file once it has been downloaded from the Google Spreadsheet, making it possible to receive data from different database models. Farmers can access the database in the field using the headset interface, and it can be displayed as an overlay on their field of view in the real world through the headset's screen. The application pairs with the glove at startup by showing an interface reporting the Bluetooth scanning result and connecting with the device through the "Connect" button. Once selected, the application establishes a connection and exchanges the required information using the GATT protocol for Bluetooth Low Energy devices, which preserves the glove battery. After that, the glove can be used to scan the bolus or the animal's ear. If the RFID code matches an entry in the database, the application retrieves all the related data, presenting it in a tabular view. Otherwise, the application shows a descriptive message (e.g., the RFID code is not included in the database). Farmers can customize the visualization by hiding columns of the table they are not interested in. By pressing the "Parameters" button, the application shows a list of all the columns in the spreadsheet, and farmers can select or deselect them.

The application allows offline work by locally caching the changes to the database and synchronizing it back on the cloud by pressing the "Update Database" button. The application notifies the farmer in case of database update issues or when the application has no locally saved data.

## Materials and methods

To evaluate the operative performances of the different systems, two sets of trials were designed. The first one was a completion of the experiments described in Todde et al.^35^ and were carried out in the laboratory to assess the complete operativity of the SMGL and to evaluate its performance of RFID tag reading and connection with ARSG in a controlled environment. The second one was carried out at the experimental sheep farm of the University of Sassari to assess the operating capabilities of the developed framework in a real farm scenario. During all the tests, a commercial RFID reader (F1 Reader produced by Datamars, Spain) was used to compare the developed systems with a conventional tool.

### Ethics approval for experimental animals and human participants

All the procedures of this research including the trials involving animals (sheep) were carried out in accordance with relevant guidelines and regulations. All animal data used in this study were collected as part of standard farming practices, where the animals were handled exclusively by the technicians of the farm. As such, no part of this research was subject to the Italian and EU legislation's approval (D.L. 26/2014 and Council Directive 2010/63/EU) or approval of an ethics committee. Moreover, based on previous research protocols the ATS Sardegna ethical committee reported that, when no healthcare investigators interact with the procedures of the study, the adopted protocol does not require an opinion from the ethics committee. Finally, informed consent was obtained from all the human participating subjects for publication of identifying information and images in an online open-access publication.

### Laboratory tests

To evaluate the operative performance of SMGL, preliminary tests were carried out in the laboratory of the Department of Agriculture of the University of Sassari. First, the activation distance of tags with the SMGL was measured. The transponders were located for 3 s at determined distances (10 cm, 5 cm, 4 cm, 3 cm, 2 cm, 1 cm), with 50 repetitions for every distance. For this test, the SMGL was disconnected from the ARSG, however, the valid activation of the transponder was confirmed by the vibration feedback of the SMGL. Second, the reading time process of the tags was measured with a stopwatch. The process ranged from the activation of the transponder at 2 cm to the complete visualization of the data in the ARSG display. The measurement was repeated 50 times for every ARSG. All the tests were performed with two types of tags, the FDX ear tag (ET) and FDX rumen bolus (RB), and with both ARSG (BT300 and HL).

### On-field tests

**Figure 3:**
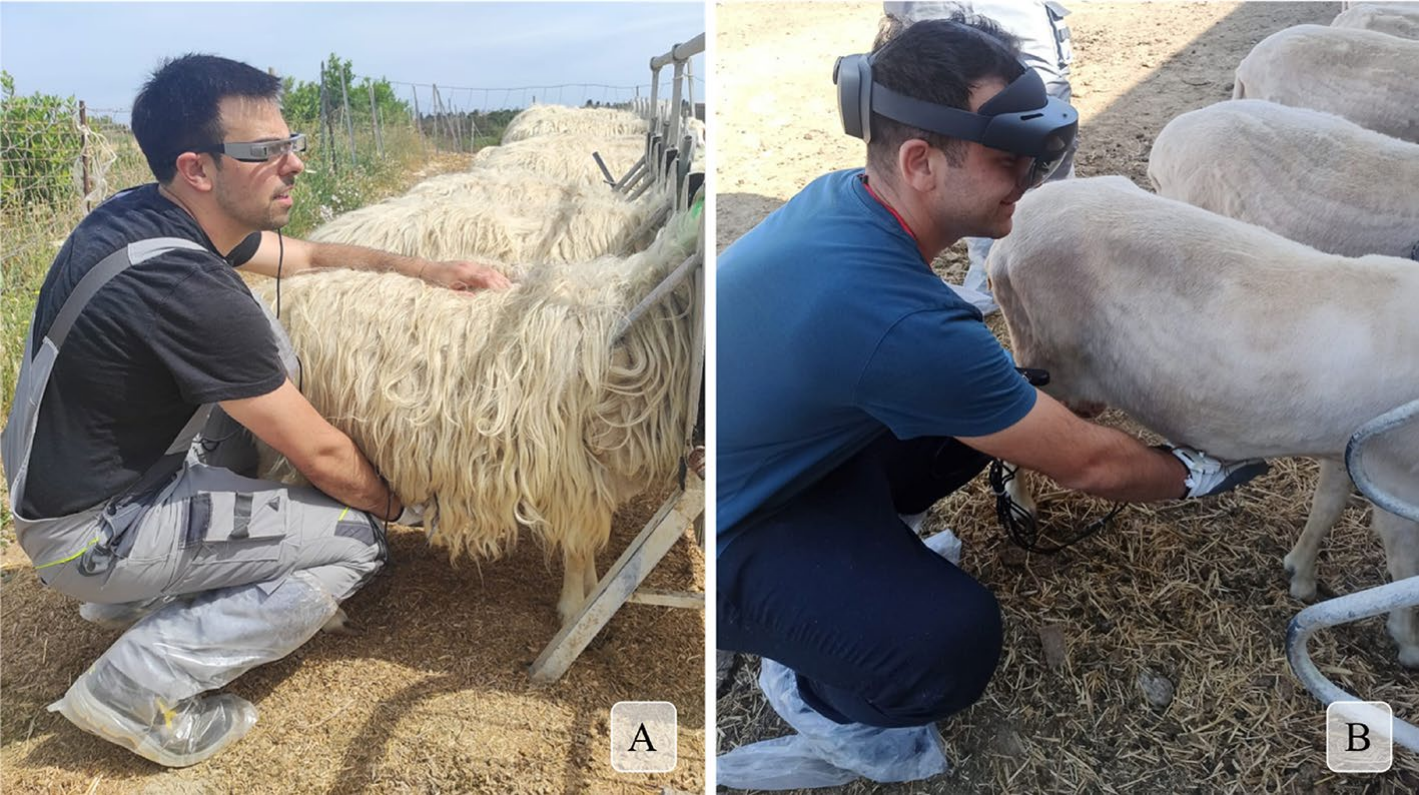
On-field tests with operators reading sheep bolus with the developed systems and consulting individual animal information through smart glasses: (**A**) operator wearing SmartGlove + Epson Moverio BT-300, (**B**) operator wearing SmartGlove + Microsoft HoloLens 2.

On-field trials were carried out to evaluate the performance of the SMGL systems for real livestock activity (Fig. 3). Specifically, the task consisted of the identification of a specific animal through the reading of the RB, and the subsequent finding of specific information in the farm database. In this case, the information to identify was the group in which the animal was collocated (A or B). A total of 18 people were recruited for the study. The group was gender balanced and consisted of students, graduates and local stakeholders. Their mean (minimum–maximum) age and body mass were 34 (22–60) years old and 66.4 (49–93) kg, respectively.

Additionally, 83% of the participants had at least a bachelor’s degree, and 59% stated that they had at least basic knowledge of AR and SG concepts and functions. All the participants reported being healthy, having normal or corrected vision with contact lenses or glasses and having no current or recent injuries that limited their normal physical ability. Before any data collection, participants were asked to complete and sign an informed consent approved by the University of Sassari. All the participants were asked to perform the task with three different types of hardware, HL connected to the SMGL, BT300 connected to the SMGL, and, finally, with the commercial RFID reader and a paper list. Five animals were placed in a rack, and the participants could choose from which side of the rack to begin the task. When the supervisor gave the signal, the operator could start to scan the animals’ RB. To verify the correct identification of the animal, the participants had to communicate to the supervisor the last three digits of the animal ID and the group in which it is collocated. The task was considered completed when all five animals were identified. During the trials, the times for each scan, the total amount of time needed to complete the task, the number of activation failures of the RFID tags and the number of wrong consultations of the database from the operator were measured. Tests were performed to evaluate the influence of the hardware type on the execution time, usability and workload. Prior to the test session, participants received a training session to become familiar with both the devices and methods. After each task simulation, they completed a set of questionnaires for the given conditions. The first questionnaires used in the research were the NASA Task Load Index (NASA-TLX), which is considered a valid tool to assess the perceived workload of a task^36,37^. This questionnaire uses six categories to assess the mental workload, measuring mental demand, temporal demand, performance, effort and frustration. Participants were asked to rate these categories on a bipolar twenty-step scale. The second questionnaire was the IBM Post Study System Usability Questionnaire (PSSUQ)^38^, in which participants are provided with different statements (in this case, 6 out of the 19 originals) and were asked how much they agree with such statements on a five-point Likert-scale (from strongly disagree to strongly agree). After completion of all the experimental conditions, the participants were asked to provide any opinion or thought about the tested devices and to select the most and least preferred system.

### Statistical analysis

For the analysis of the influence of different types of systems on operative performances, a one-way ANOVA was used. Descriptive statistics (arithmetic average, standard deviation) were calculated for each of the weighted scores of the NASA-TLX and for each category of the PSSUQ. The Kruskal‒Wallis test was used to compare the overall scores of the NASA-TLX and PSSUQ due to nonparametric data trends. RStudio (version 2022.07.2 build 576) was used to perform the statistical analysis.

## Results

### Laboratory tests

Table 2 shows the results of the activation distance for the commercial RFID reader and the SMGL with both types of transponders (ET and RB). A maximum activation distance of 5 cm resulted for both the SMGL (5% ET and 25% RB) and the commercial RFID reader (95% ET and 65% RB), with both types of transponders adopted but with different success rates. Regarding the SMGL, an acceptable success rate was observed at a distance of 3 cm (70% ET and 60% RB), while a 100% success rate was obtained at 1 cm for both types of transponders. The F1 reader obtained similar results with an acceptable success rate (95% for ET and 65% for RB) at 5 cm, and a secure activation of the transponders, both ET and RB, at 2 cm.

**Table 2:** Comparative results between the SMGL and the F1 reader of transponder activation in relation to the distance between transceivers and transponders, ear tag (ET) and rumen bolus (RB). N = 1200.

Distance (cm)	Success rate (%)			
	SMGL			F1 reader
	ET	RB	ET	RB
10	0.0	0.0	0.0	0.0
5	5.0	25.0	95.0	65.0
4	10.0	15.0	100.0	65.0
3	70.0	60.0	100.0	65.0
2	100.0	60.0	100.0	100.0
1	100.0	100.0	100.0	100.0

In Table 3, the results of the reading process time are presented. The time framework recorded with BT300 was 2.16 s with ET, and 2.22 s with RB, while for HL, it was 4.30 s with ET, and 3.80 s with RB. HL showed a higher variability in the reading process time in comparison with BT300, having a difference between the minimum and maximum time of 7.90 s for HL, and 2.87 s for BT300.

**Table 3:** Average time of transponder reading and data visualization in the BT300 and HL displays, Ear tag (ET) and rumen bolus (RB). SD = standard deviation. N = 200.

	Reading process time (s)			
	BT300			HL
	ET	RB	ET	RB
Mean	2.16	2.22	4.30	3.80
SD	0.37	0.27	2.02	2.11
Min	1.59	1.23	2.23	2.24
Max	3.00	2.86	8.62	10.13

### On field test

The type of system used for animal identification affected the operating performances with statistical relevance (*p* < 0.001). Participants performed the task in a shorter time with the conventional system (mean = 59.79, SD = 15.40) than with the ARSG system (mean = 82.72, SD = 32.81) or with the MRSG system (mean = 98.33, SD = 35.04). The same results were obtained with the average time per tag reading with the conventional method, which was faster (mean = 12.45, SD = 2.93), followed by the ARSG system (mean = 16.86 SD = 4.34) and finally the MRSG system (mean = 21.32, SD = 7.39). With all the tested systems, no errors occurred (Table 4).

**Table 4:** Means and standard deviation of times (in seconds) for the whole task and for each animal identification with the three systems tested. N = 270.

	F1 reader + paper list	BT300 + SMGL	HL + SMGL
Task completion time	59.79 ± 15.40^a^	82.72 ± 32.81^ab^	98.33 ± 35.04^c^
Time per head	12.45 ± 2.93^a^	16.86 ± 4.34^ab^	21.32 ± 7.39^c^

### Questionnaires results

The results of the analyses of the NASA-TLX did not highlight significant differences in the type of system used for every category of the questionnaire (Table 5). However, differences in the scores between each type of system were appreciable. Specifically, the mental demand, temporal demand and frustration levels were lower in the BT300 + SMGL and HL + SMGL systems than in the conventional system (F1 reader + paper list), while physical demand and performance satisfaction showed no particular differences. Finally, the overall workload score was higher for the conventional system (29.72), followed by the HL + SMGL system (27.56) and the BT300 + SMGL system (25.19).

**Table 5:** Summary of the NASA-TLX subscale scores with mean and standard deviation.

Category	F1 reader + Paper list	BT300 + SMGL	HL + SMGL
Mental demand	21.67 ± 17.92	17.50 ± 19.93	16.71 ± 18.73
Physical demand	9.00 ± 8.91	10.11 ± 7.90	11.53 ± 12.13
Temporal demand	24.33 ± 21.49	18.72 ± 20.79	17.94 ± 20
Performance	20.71 ± 21.21	19.89 ± 20.79	25.33 ± 24.79
Effort	9.11 ± 10.35	9.50 ± 13.05	10.41 ± 10.30
Frustration	15.75 ± 14.88	3.20 ± 1.81	13 ± 9.41
Overall workload	29.72 ± 21.18	25.19 ± 16.82	27.56 ± 17.44

**Figure 4:**
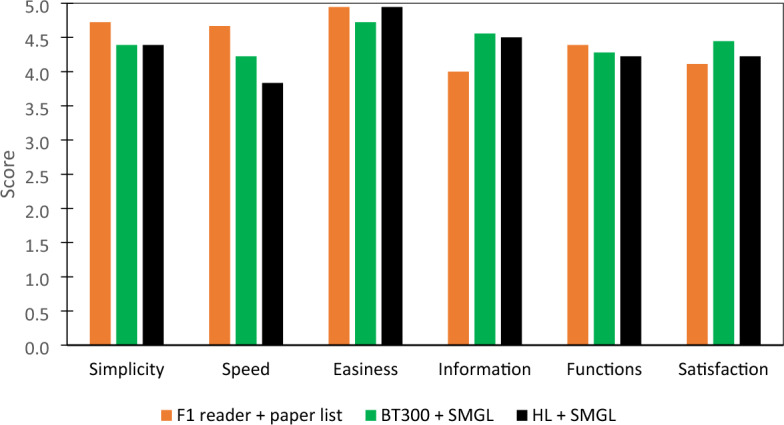
Mean score by type of system (F1 reader + paper list, BT300 + SMGL, HL + SMGL) per question of the IBM-PSSUQ questionnaire.

Similar to the outcomes of the NASA-TLX questionnaire, the results obtained with the PSSUQ showed no significant differences. However, appreciable variances of scores in some categories of the questionnaire can be found (Fig. 4). Regarding the speed of the system, the conventional systems showed the best scores (4.67) in comparison to the BT300 + SMGL system (4.22) and the HL + SMGL system (3.83). Moreover, the easiness of the information findings showed substantial differences between the systems, where BT300 + SMGL and HL + SMGL obtained higher scores (4.56 and 4.50, respectively) than the conventional methods (4.00).

## Discussion

This study aimed to develop a functional framework that allows the visualization of specific animal data directly on-field thanks to the connection between animal RFID tags and smart glasses for augmented reality. The individuation of specific information on an animal in a livestock farm could improve specific on-farm activities, such as animal grouping based on the number of lambs or the average milk yield^39^. Moreover, the operative performances of the systems developed were evaluated during laboratory tests and on-field livestock management operations. In addition, as the system is capable of connecting with different types of ARSG, the suitability of various types of SG and different levels of AR technology in the agricultural environment was evaluated. The laboratory tests confirmed the complete capability of the SMGL system to function in a controlled environment, showing a performance similar to that of a commercial RFID reader. These outcomes allowed the upgrade of the SMGL from a TRL-3 (analytical and experimental critical function and/or characteristic proof of concept) to a TRL-4 prototype (component and/or breadboard functional verification in laboratory environment). Moreover, the on-field task showed that the developed systems can be used as a possible tool for the identification of livestock animals in agricultural environments, upgrading the TRL of the device to level 5 (component and/or breadboard critical function verification in a relevant environment). However, the use of ARSG led to longer operative times compared to conventional systems, in coherence with similar studies made in other industry sectors (e.g., assembly) where the use of AR systems showed a longer assembly time compared to the use of paper, video, or tablet systems^40,41^. However, in this study, the longer operative time in the identification task is imputable to the lower success rate of the tag activation of the SMGL in comparison to the conventional RFID reader. Additionally, the low level of optimization is underlined by the difference, in terms of time per reading, between the laboratory (Table 3) and the on-field trials (Table 4). In addition, it must be considered that for the conventional methods, paper lists have to be prepared before the start of operations, while both developed systems can be considered ready-to-go. As observed by Drouot et al.^42^, the level of familiarity of the user with new technology may have an impact on user performance. In fact, even if the participants received a brief training, most of them (12 out of 18) reported no, or low, previous knowledge of AR or SG. Moreover, as confirmed by the NASA-TLX scores, the animal identification task was straightforward, while the AR systems showed better results in complex operations^43,44^. A possible explanation for the difference between BT300 + SMGL and HL + SMGL, in terms of operative performances, could be related to the spatialization of the information of the MR system. In fact, while in assisted reality systems, the display with all the information is always visible in front of the operator, in the MR system, information are in a precise position of the real environment and, without familiarity with this technology, can be difficult to find^45^. Additionally, the different scores of mental workloads could also suggest that the use of ARSG improve the levels of situational awareness (the ability to perceive, understand, and effectively respond to a specific situation), which is one of the most relevant elements for the evaluation of operator safety^46^. Furthermore, the scores regarding the statements for “simplicity of use” and “speed of the system” in the IBM–PSSUQ were higher for the conventional system. In fact, the high level of familiarity of participants with the conventional tools allowed them to complete the task in a shorter time. Nevertheless, as shown by the “easiness of finding information” statement, the ARSG systems permitted a faster localization of the information. The elements discussed previously (i.e., low optimization level of the SMGL, lack of familiarity with the AR technology and the simplicity of the task) may also have contributed to the low difference among the scores of the three different systems in the poststudy questionnaires. However, participants underlined the advantages of AR systems in comparison to conventional systems, such as the benefits of voice commands and the possibility of a hands-free operation. The participants were also asked to select a preferred and least preferred system, and 11 out of 18 selected HL + SMGL, 7 out of 18 selected BT300 + SMGL and no one selected the conventional method, which was indicated as the least preferred by 12 out of 18 participants.

## Conclusion

In this study, the development and testing of a smart RFID wearable reader that aims to bridge the technological gap between the electronic identification of livestock animals and real-time access to individual information was analyzed. The device, named SmartGlove, was developed by combining the functionalities of an RFID reader with smart glasses for augmented reality. In addition, to connect SMGL and ARSG, two specific applications for Microsoft HoloLens 2 and Epson Moverio BT-300 were coded. The laboratory and on-field tests underlined promising operating performances allowed the upgrade, in terms of TRL of the device from level 3 to level 5. The on-field trials allowed stakeholders to visualize animal information directly on-farm, and superimposed on the specific animal, in a short time interval. The participants’ feedback confirmed a low cognitive impact and a high usability level for the use of the SMGL connected to ARSG. Finally, the operators underlined the preference for SMGL systems over conventional systems for animal identification and the consequent visualization of animal data. However, the SMGL is still in a prototype phase, and further improvements are needed, focusing on the miniaturization of the hardware and the design of a more ergonomic and comfortable tool shape. Finally, future works will also focus on upgrading the ARSG software, with a completer and more intuitive user interface and a more comprehensive and automated system for the precision management of animal data.

## Data Availability

The datasets generated during the current study are available from the corresponding author on reasonable request.
